# The complete chloroplast genome of *Malva cathayensis* M.G.Gilbert, Y.Tang & Dorr 2007 and its phylogenetic analysis

**DOI:** 10.1080/23802359.2025.2466580

**Published:** 2025-02-23

**Authors:** Shuming Zhang, Kaihua Zhang, Yuting Jiao, Junfei Liu, Weihan Yuan, Liqiang Wang

**Affiliations:** College of Pharmacy, Heze University, Heze, PR China

**Keywords:** Chloroplast genome, Malvaceae, *Malva cathayensis*, phylogenetic analysis

## Abstract

*Malva cathayensis*, a wild medicinal and edible Malvaceae species, lacked genomic data until now. In this study, we presented its first complete chloroplast genome (158,793 bp), featuring a quadripartite structure: 87,215 bp LSC, 20,766 bp SSC, and two 25,406 bp IRs. The genome contains 129 genes (85 protein-coding, 36 tRNA, 8 rRNA) with 37.1% GC content. Phylogenetic analysis revealed two *Malva clades*, with *M. cathayensis* grouping alongside *M. crispa, M. verticillata*, and *M. parviflora*. This study provides essential molecular data for *Malva*'s evolutionary relationships and diversification, enabling future comparative genomic research in this genus.

## Introduction

*Malva* is a herbaceous plant genus in the Malvaceae family, with about 30 species found in Africa and Eurasia across temperate, subtropical, and tropical climate zones (Rasheed et al. 2017). These species have been used in traditional medicine since antiquity. The leaves and flowers of *Malva* plants contain various bioactive compounds, including polysaccharides, coumarins, flavonoids, polyphenols, vitamins, terpenes, and tannins. The biological properties of these compounds include moderate antimicrobial, high anti-inflammatory, wound healing, strong antioxidant, and anticancer activities (Sharifi-Rad et al. 2020).

**Figure 1.**
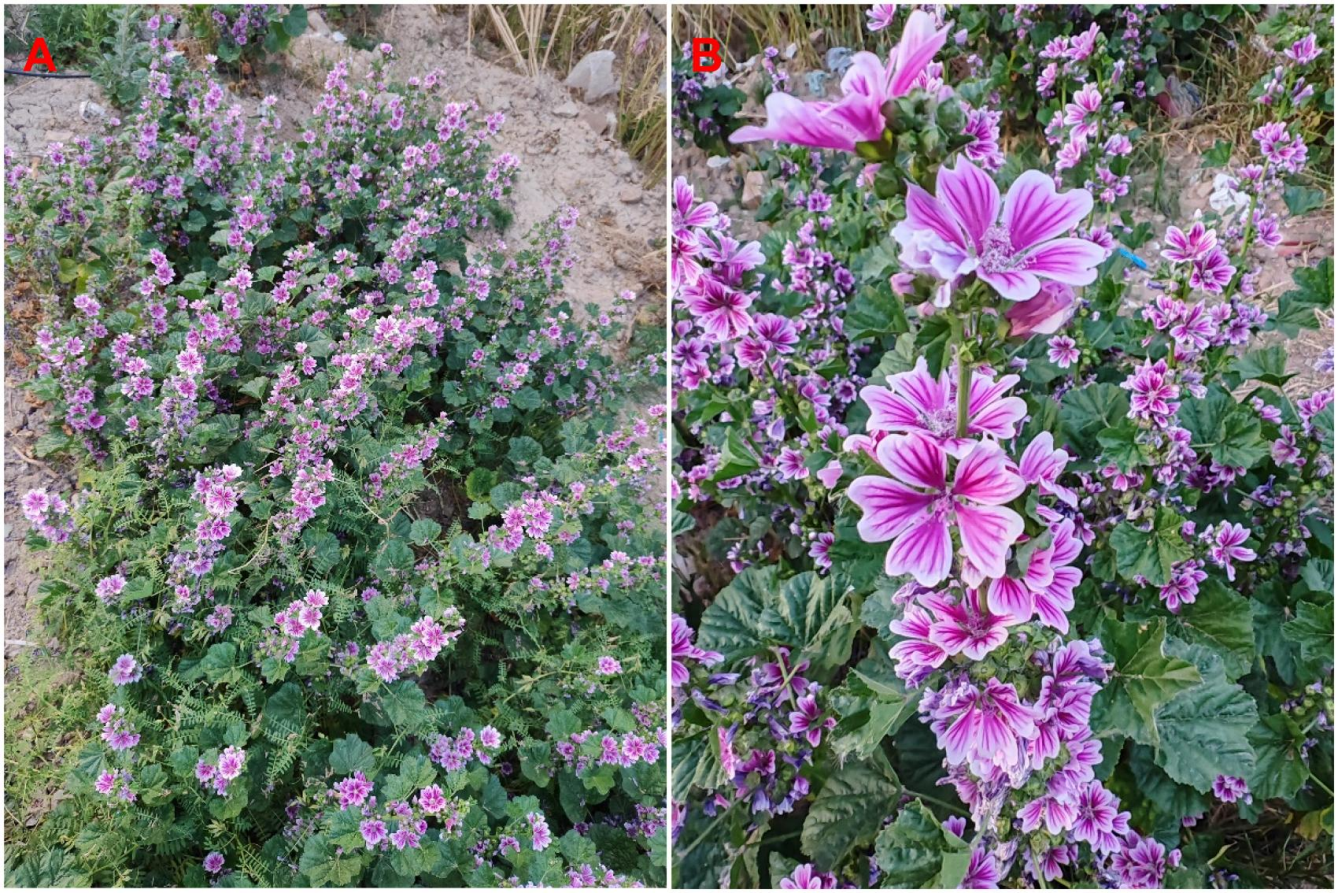
Panoramic (A) and detailed (B) photos of *Malva cathayensis*. Liqiang Wang photographed the plant located at 35°16′10″N, 115°27′56″E. Main identifying traits of the species: erect, biennial, or perennial herb, 50–90 cm tall, with multiple branches and sparse, coarse hairs. The leaves are round-heart or kidney-shaped and have 5–7 rounded, toothed lobes. Both sides are mostly hairless, with short hairs along the veins. Flowers are clustered in groups of 3–11, with three tiny, elongated bracts that are sparsely pilose. Flowers are purple-red or white, about 3.5–4 cm in diameter, with five 2 cm long spoon-shaped petals, slightly notched at the apex and barbed at the base. The fruit is flat and round, with 9–11 kidney-shaped mericarps covered in soft hairs. Seeds are black-brown, kidney-shaped, and 2 mm long. Flowering lasts from May until October.

*Malva cathayensis* M.G.Gilbert, Y.Tang & Dorr 2007 (Gilbert *et al*. 2007), also referred to as *Malva cavanillesiana* Raizada 1976, is an erect, much-branched, biennial to perennial plant that grows 50–90 cm tall (Figure 1). It is harvested from the wild for medicinal purposes, as well as for food and materials. The species also finds use as an ornamental plant, particularly in China and India (Gilbert et al. 2007). *Malva cathayensis* is classified as a cadmium (Cd) accumulator or a nonstandard Cd-hyperaccumulator (Zhang et al. 2010). However, little genetic information is available about *M. cathayensis*. This study reports the first complete sequencing and characterization of its chloroplast genome. These results provide essential genomic resources for species identification, population genetics, and germplasm exploitation.

## Materials and methods

*Malva cathayensis* specimens were collected from the Peony District in Heze City, Shandong Province, China (35°16′10″N, 115°27′56″E). The specimen was deposited in the Heze University Herbarium under the specimen number HZ2101005 (contact: Liqiang Wang, lys832000@163.com).

Total genomic DNA was extracted using a plant genomic DNA kit (Tiangen Biotech, Beijing, China). The DNA was fragmented to approximately 300 bp to create a 150 bp paired-end library, which was sequenced on the Illumina NovaSeq 6000 platform (Illumina, San Diego, CA) by Wuhan Benagen Technology Company Limited (Wuhan, China). Raw reads were quality-checked using FastQC (https://www.bioinformatics.babraham.ac.uk/projects/fastqc/), and low-quality reads were filtered with Trimmomatic (Bolger et al. 2014). The trimmed reads were assembled using GetOrganelle v1.1.7 (Jin et al. 2020). The assembled genome was annotated with CPGAVAS2.0 (Shi et al. 2019) and visualized in CPGView (Liu et al. 2023). We determined the genome assembly reliability by estimating sequencing depth using minimap2 (Li 2018) and samtools (Li et al. 2009).

For phylogenetic analysis of *Malva cathayensis*, six other *Malva* chloroplast genomes were downloaded from GenBank, with *Abelmoschus esculentus* (Malvaceae) serving as an outgroup. MAFFT was used to align the complete chloroplast genomes of seven *Malva* species and the outgroup (Katoh and Standley 2013). Phylogenetic analysis was performed in IQ-TREE v1.6.8 (Nguyen et al. 2015) using the maximum-likelihood (ML) approach and the K3Pu + F + I nucleotide substitution model selected by ModelFinder (Kalyaanamoorthy et al. 2017).

## Results

**Figure 2.**
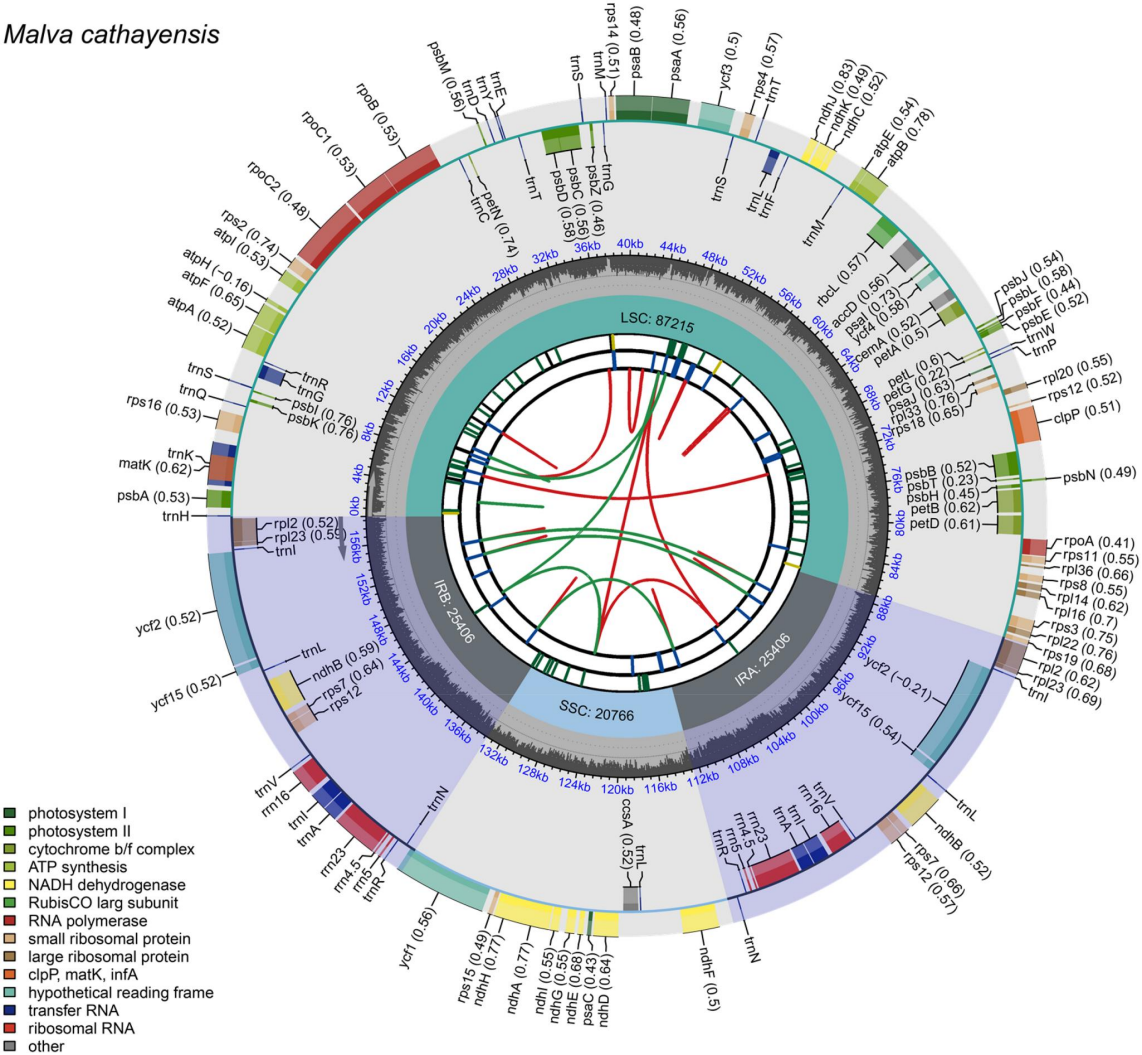
A schematic map depicting the overall features of the *Malva cathayensis* chloroplast genome. From the center outward: the first track represents dispersed repeats, the second track displays long tandem repeats as short blue bars, and the third track shows short tandem repeats (microsatellites) as color-coded bars. The fourth track exhibits the small single-copy (SSC), inverted repeat (IRa and IRb), and large single-copy (LSC) regions. The fifth track plots the GC content of the genome, while the sixth track displays gene locations. The functional type of the genes is shown in the bottom left corner. For protein-coding genes, letters after gene names suggest functional subunits or family members, while numbers denote gene variants or functional differentiation. For ribosomal genes, numbers after gene names represent rRNA size in Svedberg units. Letters after gene names for tRNA genes denote the amino acids that the tRNA recognizes. For genes with unknown function, the numbers after the gene names correspond to distinct hypothetical coding genes.

The whole genome DNA was successfully sequenced, yielding approximately 16.2 GB of raw data (fastq format). The assembled *M. cathayensis* chloroplast genome is a circular DNA molecule with a total length of 158,793 bp. Mapping results verified the fidelity of genome assembly, with an average sequencing depth of 1895.35× and a minimum depth of 577× (Figure S1). The genome exhibits the typical quadripartite structure, with a large single-copy (LSC) region (88,302 bp), a small single-copy (SSC) region (20,766 bp), and a pair of inverted repeat (IR) regions (25,406 bp each). The overall GC content is 37.1%, with the IR regions having a higher content (42.96%) and the LSC and SSC regions having lower content (34.93% and 32.02%, respectively). The genome encodes 129 genes, including 85 protein-coding genes, 36 tRNA genes, and eight rRNA genes (Figure 2). Fourteen protein-coding genes are cis-splicing, including *rps*16, *atp*F, *rpo*C1, *ycf*3, *clp*P, *pet*B, *pet*D, *rpl*2 (×2), *ycf*15 (×2), *ndh*B (×2), and *ndh*A (Figure S2A). Two of these genes, *ycf*3 and *clp*P, encompass two introns. The *rps*12 gene is trans-splicing and includes two introns (Figure S2B). Five tRNA genes (*trn*K-UUU, *trn*G-UCC, *trn*L-UAA, *trn*I-GAU, and *trn*A-UGC) possess one intron.

**Figure 3.**
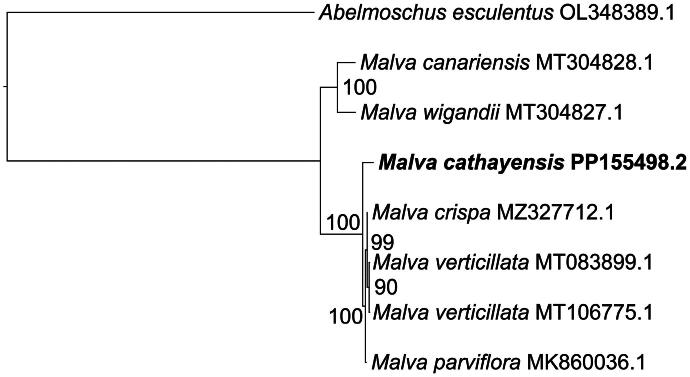
The maximum-likelihood phylogenetic tree featuring *Malva cathayensis.*

Phylogenetic analysis using the ML method revealed that all *Malva* species clustered together two distinct clades (Figure 3). *Malva canariensis* and *M. wigandii* formed a monophyletic clade with 100% bootstrap support, while *M. cathayensis* grouped with *M. crispa*, *M. verticillata*, and *M. parviflora* in another monophyletic clade, with bootstrap values exceeding 90%.

The tree was constructed using the complete chloroplast genome sequences of *M. cathayensis* (PP155498.2, this study) and five other *Malva* species, including *M. canariensis* (MT304828.1), *M. wigandii* (MT304827.1) (García-Mir et al. 2021), *M. crispa* (MZ327712.1), *M. verticillata* (MT083899.1, MT106775.1) (Li et al. 2020; Wang et al. 2020), and *M. parviflora* (MK860036.1) (Abdullah et al. 2021). *Abelmoschus esculentus* (OL348389.1) (Liu et al. 2023) served as the outgroup. Bootstrap support values were calculated from 1000 replicates and are shown at each node. *Malva cathayensis* is highlighted in bold in the phylogenetic tree.

## Conclusions and discussion

Our study presents the first complete chloroplast genome and the phylogenetic analysis of *M. cathayensis*, enhancing our understanding of its evolutionary relationships within the genus. It also provides a valuable genetic resource for future *Malva* genus research.

Comparative analyses showed that the chloroplast genomes of *Malva* species are highly conserved in structure, length, and gene content. The chloroplast genome of *M. cathayensis* has a quadripartite structure similar to that of other *Malva* species. The chloroplast genome of *M. cathayensis* is also comparable to other *Malva* species in terms of genome length and gene count, with a range of 158.1–158.5 kb and 129–131 genes, respectively (Li et al. 2020; Wang et al. 2020; Abdullah et al. 2021; García-Mir et al. 2021).

The taxonomy and systematics of the genus *Malva* remain ambiguous and challenging due to the high level of homoplasy in morphological traits. A common approach divides *Malva* into two sections based on different criteria. Dalby (1968) proposed floral structure-based division. Ray (1995, 1998) described classification based on ITS molecular markers, fruit morphology, and seed structure. Jedrzejczyk and Rewers (2020) outlined the use of genome size estimation and ISSR molecular markers for categorization. *Malva parviflora* and *M. verticillata* are grouped in the same section of the phylogenetic tree created using chloroplast genome data, consistent with previous classifications based on different criteria (Ray 1995, 1998; Jedrzejczyk and Rewers 2020). The grouping of *M. cathayensis*, *M. crispa*, *M. parviflora*, and *M. verticillata* in one section and *M. canariensis* and *M. wigandii* in another separate section offers valuable insights for resolving the taxonomy of *Malva*.

## Supplementary Material

SMZhang_250131_traced.docx

## Data Availability

The chloroplast genome sequence has been deposited in GenBank (https://www.ncbi.nlm.nih.gov/genbank/) with the accession number of PP155498.2 (https://www.ncbi.nlm.nih.gov/nuccore/PP155498.2). The associated BioProject, Bio-Sample, and SRA numbers are PRJNA928567, SAMN42702630, and SRR29906881, respectively.

## References

[CIT0001] Abdullah , Mehmood F, Shahzadi I, Ali Z, Islam M, Naeem M, Mirza B, Lockhart PJ, Ahmed I, Waheed MT, et al. 2021. Correlations among oligonucleotide repeats, nucleotide substitutions, and insertion–deletion mutations in chloroplast genomes of plant family Malvaceae. J Syst Evol. 59(2):388–402. doi:10.1111/jse.12585.

[CIT0002] Bolger AM, Marc L, Bjoern U. 2014. Trimmomatic: a flexible trimmer for Illumina sequence data. Bioinformatics. 30(15):2114–2120. doi:10.1093/bioinformatics/btu170. 24695404 PMC4103590

[CIT0003] Dalby DH. 1968. *Malva* L. In: Tutin TG, Heywood VH, Burges NA, Moore DM, Valentine DH, Walters SM, Weeb DA. Flora Europaea. Rosaceae to Umbelliferae. Cambridge (UK): Cambridge University Press. 2: 249–251.

[CIT0004] García-Mir L, Ojeda DI, Fuertes-Aguilar J. 2021. The complete chloroplast genome of *Malva wigandii* (Alef.) M.F. Ray (Malvaceae, Malvoideae). Mitochondrial DNA B Resour. 6(3):1181–1182. doi:10.1080/23802359.2021.1902409. 33796779 PMC7995849

[CIT0005] Gilbert MG, Tang Y, Dorr LJ. 2007. Malva cathayensis M.G. Gilbert, Y. Tang & Dorr. Flora China. 12:266.

[CIT0006] Jedrzejczyk I, Rewers M. 2020. Identification and genetic diversity analysis of edible and medicinal *Malva* species using flow cytometry and ISSR molecular markers. Agronomy. 10(5):650. doi:10.3390/agronomy10050650.

[CIT0007] Jin JJ, Yu WB, Yang JB, Song Y, dePamphilis CW, Yi TS, Li DZ. 2020. GetOrganelle: a fast and versatile toolkit for accurate *de novo* assembly of organelle genomes. Genome Biol. 21(1):241. doi:10.1186/s13059-020-02154-5. 32912315 PMC7488116

[CIT0008] Kalyaanamoorthy S, Minh BQ, Wong TKF, von Haeseler A, Jermiin LS. 2017. ModelFinder: fast model selection for accurate phylogenetic estimates. Nat Methods. 14(6):587–589. doi:10.1038/nmeth.4285. 28481363 PMC5453245

[CIT0009] Katoh K, Standley DM. 2013. MAFFT multiple sequence alignment software version 7: improvements in performance and usability. Mol Biol Evol. 30(4):772–780. doi:10.1093/molbev/mst010. 23329690 PMC3603318

[CIT0010] Li H, Handsaker B, Wysoker A, Fennell T, Ruan J, Homer N, Marth G, Abecasis G, Durbin R, 1000 Genome Project Data Processing Subgroup. 2009. The sequence alignment/map format and SAMtools. Bioinformatics. 25(16):2078–2079. doi:10.1093/bioinformatics/btp352. 19505943 PMC2723002

[CIT0011] Li H. 2018. Minimap2: pairwise alignment for nucleotide sequences. Bioinformatics. 34(18):3094–3100. doi:10.1093/bioinformatics/bty191. 29750242 PMC6137996

[CIT0012] Li R, Liu J, Xu L, Duan B, Qian J. 2020. The complete chloroplast genome of *Malva verticillata* (Malvaceae). Mitochondrial DNA B Resour. 5(2):1609–1610. doi:10.1080/23802359.2020.1745106.

[CIT0013] Liu S, Ni Y, Li J, Zhang X, Yang H, Chen H, Liu C. 2023. CPGView: a package for visualizing detailed chloroplast genome structures. Mol Ecol Resour. 23(3):694–704. doi:10.1111/1755-0998.13729. 36587992

[CIT0014] Liu Y, Wang J, Bai Y, Zhang T, Shi D, Liu Z, Jiang L, Ye L. 2023. The whole chloroplast genome in *Abelmoschus esculentus* L. Moench. N Z J Crop Hortic Sci. 51(1):123–135. doi:10.1080/01140671.2021.1960568.

[CIT0015] Nguyen LT, Schmidt HA, von Haeseler A, Minh BQ. 2015. IQ-TREE: a fast and effective stochastic algorithm for estimating maximum-likelihood phylogenies. Mol Biol Evol. 32(1):268–274. doi:10.1093/molbev/msu300. 25371430 PMC4271533

[CIT0016] Rasheed HU, Nawaz H, Rehman R, Mushtaq A, Khan S, Azeem W. 2017. Little mallow: a review of botany, composition, uses and biological potentials. Int J Chem Biochem Sci. 12:157–161.

[CIT0017] Ray MF. 1995. Systematics of *Lavatera* and *Malva* (Malvaceae, Malveae)—a new perspective. Plant Syst Evol. 198(1–2):29–53. doi:10.1007/BF00985106.

[CIT0018] Ray MF. 1998. New combinations in *Malva* (Malvaceae: Malveae). Novon. 8(3):288–295. doi:10.2307/3392022.

[CIT0019] Sharifi-Rad J, Melgar-Lalanne G, Javier Hernández-Álvarez A, Taheri Y, Shaheen S, Kregiel D, Antolak H, Pawlikowska E, Brdar-Jokanović M, Rajkovic J, et al. 2020. *Malva* species: insights on its chemical composition towards pharmacological applications. Phytother Res. 34(3):546–567. doi:10.1002/ptr.6550. 31713320

[CIT0020] Shi L, Chen H, Jiang M, Wang L, Wu X, Huang L, Liu C. 2019. CPGAVAS2, an integrated plastome sequence annotator and analyzer. Nucleic Acids Res. 47(W1):W65–W73. doi:10.1093/nar/gkz345. 31066451 PMC6602467

[CIT0021] Wang L, Cai B, Li J, Yang Y. 2020. The complete chloroplast genome sequence of *Malva verticillate*. Mitochondrial DNA B Resour. 5(2):1669–1670. doi:10.1080/23802359.2020.1742602. PMC7510816 33366921

[CIT0022] Zhang S, Chen M, Li T, Xu X, Deng L. 2010. A newly found cadmium accumulator—*Malva sinensis* Cavan. J Hazard Mater. 173(1–3):705–709. doi:10.1016/j.jhazmat.2009.08.142. 19767144

