# Supplementary material for: The complete chloroplast genome of Malva cathayensis M.G.Gilbert, Y.Tang & Dorr 2007 and its phylogenetic analysis
Source: Mitochondrial DNA B Resour. 2025 Feb 23;10(3):229–32. doi: 10.1080/23802359.2025.2466580 (PMC11852229; doi:10.1080/23802359.2025.2466580)
Supplement: SMZhang_250131_traced.docx [file TMDN_A_2466580_SM3206.docx]

# Title

The complete chloroplast genome of Malva cathayensis M.G.Gilbert, Y.Tang & Dorr 2007 and its phylogenetic analysis

# Author and affiliation

Shuming Zhang, Kaihua Zhang, Yuting Jiao, Junfei Liu, Weihan Yuan, Liqiang Wang^*^

College of Pharmacy, Heze University, Heze 274015, Shandong Province, P. R. China

# E-mails:

Shuming Zhang: 2334607779@qq.com

Kaihua Zhang: 2670810146@qq.com

Yuting Jiao: 303749906@qq.com

Junfei Liu: 3089295653@qq.com

Weihan Yuan: 1970371544@qq.com

Liqiang Wang: [lys832000@163.com](mailto:lys832000@163.com)

# *Correspondence:

Liqiang Wang: [lys832000@163.com](mailto:lys832000@163.com)

# Abstract

*Malva cathayensis* M.G.Gilbert, Y.Tang & Dorr 2007, a species in the Malvaceae family, is traditionally harvested from the wild for medicinal uses, as well as food and materials. However, little is known about this species, particularly its genetic information. In this study, we report the first complete chloroplast genome of *M*. *cathayensis*, which is 158,793 bp long. The genome consists of a large single-copy (LSC) region of 87,215 bp, a small single-copy (SSC) region of 20,766 bp, and two inverted repeat (IR) regions of 25,406 bp each. It encodes 129 genes, including 85 protein-coding genes, 36 tRNA genes, and 8 rRNA genes, with a 37.1% GC content. Phylogenetic analysis using the maximum likelihood method revealed that six *Malva* species form two distinct clades, with *M*. *cathayensis*, *M*. *crispa*, *M*. *verticillata*, and *M*. *parviflora* clustering into a monophyletic group. This study adds vital genetic data to our understanding of diversification mechanisms in the *Malva* genus.

# Keywords

Chloroplast genome, Malvaceae, *Malva cathayensis*, Phylogenetic analysis

# Introduction

*Malva* is a herbaceous plant genus in the Malvaceae family, with about thirty species found in Africa and Eurasia across temperate, subtropical, and tropical climate zones (Hafiza *et al*. 2017). These species have been used in traditional medicine since antiquity. The leaves and flowers of *Malva* plants contain various bioactive compounds, including polysaccharides, coumarins, flavonoids, polyphenols, vitamins, terpenes, and tannins. The biological properties of these compounds include moderate antimicrobial, high anti-inflammatory, wound healing, strong antioxidant, and anticancer activities (Javad *et al*. 2019).

*Malva cathayensis* M.G.Gilbert, Y.Tang & Dorr 2007 (Gilbert *et al*. 2007), also referred to as *Malva cavanillesiana* Raizada 1976, is an erect, much-branched, biennial to perennial plant that grows 50-90 cm tall (Figure 1). It is harvested from the wild for medicinal purposes, as well as for food and materials. The species also finds use as an ornamental plant, particularly in China and India (Gilbert *et al*. 2007). *Malva cathayensis* is classified as a cadmium (Cd) accumulator or a non-standard Cd-hyperaccumulator (Zhang *et al*. 2010). However, little genetic information is available about *M*. *cathayensis*. This study reports the first complete sequencing and characterization of its chloroplast genome. These results provide essential genomic resources for species identification, population genetics, and germplasm exploitation.


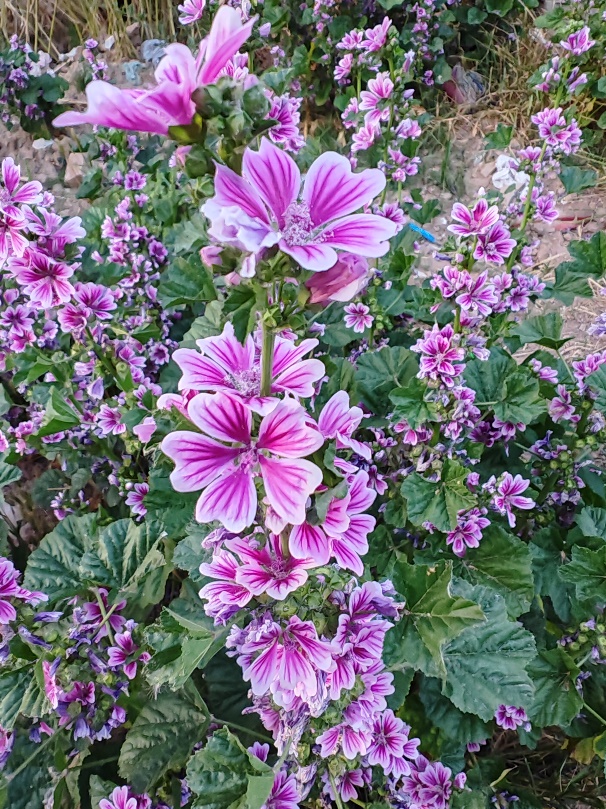

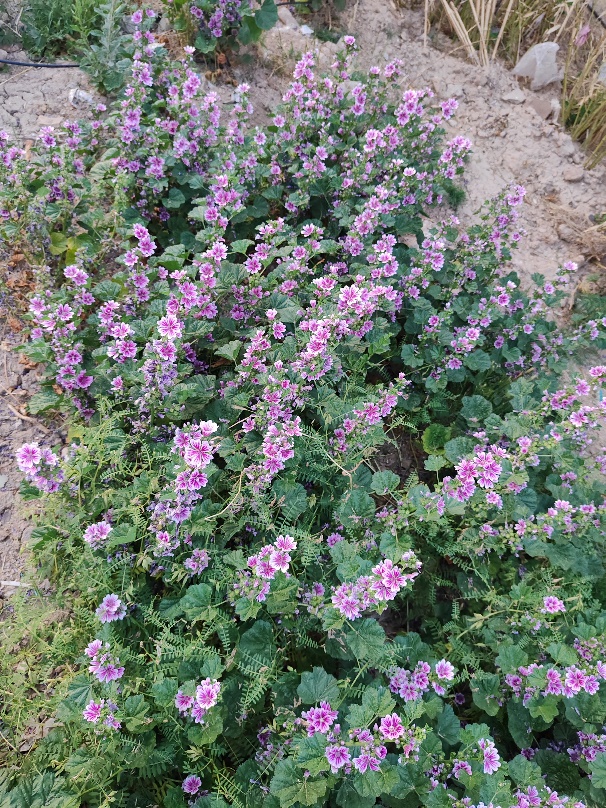


**B**

**A**

**Figure 1.** Panoramic (A) and detail (B) photos of *Malva cathayensis*. Liqiang Wang photographed the plant located at 35^o^16’10’’N, 115^o^27’56’’E. Main identifying traits of the species: Erect, biennial or perennial herb, 50–90 cm tall, with multiple branches and sparse, coarse hairs. The leaves are round-heart or kidney-shaped and have 5-7 rounded, toothed lobes. Both sides are mostly hairless, with short hairs along the veins. Flowers are clustered in groups of three to eleven, with three tiny, elongated bracts that are sparsely pilose. Flowers are purple-red or white, about 3.5-4 cm in diameter, with five 2 cm long spoon-shaped petals, slightly notched at the apex and barbed at the base. The fruit is flat and round, with 9-11 kidney-shaped mericarps covered in soft hairs. Seeds are black-brown, kidney-shaped, and 2 mm long. Flowering lasts from May until October.

# Materials and Methods

*Malva cathayensis* specimens were collected from the Peony District in Heze City, Shandong Province, China (35°16'10''N, 115°27'56''E). The specimen was deposited in the Heze University Herbarium under the specimen number HZ2101005 (contact: Liqiang Wang, lys832000@163.com).

Total genomic DNA was extracted using a plant genomic DNA kit (Tiangen Biotech, Beijing, China). The DNA was fragmented to approximately 300 bp to create a 150 bp paired-end library, which was sequenced on the Illumina NovaSeq 6000 platform (Illumina, San Diego, CA) by Wuhan Benagen Technology Company Limited (Wuhan, China). Raw reads were quality-checked using FastQC (https://www.bioinformatics.babraham.ac.uk/projects/fastqc/), and low-quality reads were filtered with Trimmomatic (Bolger *et al*. 2014). The trimmed reads were assembled using GetOrganelle v1.1.7 (Jin *et al*. 2020). The assembled genome was annotated with CPGAVAS2.0 (Shi *et al*. 2019) and visualized in CPGView (Liu *et al*. 2023a). We determined the genome assembly reliability by estimating sequencing depth using minimap2 (Li 2018) and samtools (Li *et al*. 2009).

For phylogenetic analysis of *Malva cathayensis*, six other *Malva* chloroplast genomes were downloaded from GenBank, with *Abelmoschus esculentus* (Malvaceae) serving as an outgroup. MAFFT was used to align the complete chloroplast genomes of seven *Malva* species and the outgroup (Katoh and Standley, 2013). Phylogenetic analysis was performed in in IQ-TREE v1.6.8 (Nguyen *et al*. 2015) using the maximum likelihood (ML) approach and the K3Pu+F+I nucleotide substitution model selected by ModelFinder (Kalyaanamoorthy *et al*. 2017).

# Results

The whole genome DNA was successfully sequenced, yielding approximate 16.2 GB of raw data (fastq format). The assembled *M. cathayensis* chloroplast genome is a circular DNA molecule with a total length of 158,793 bp. Mapping results verified the fidelity of genome assembly, with an average sequencing depth of 1895.35× and a minimum depth of 577× (Figure S1). The genome exhibits the typical quadripartite structure, with a large single-copy (LSC) region (88,302 bp), a small single-copy (SSC) region (20,766 bp), and a pair of inverted repeat (IR) regions (25,406 bp each). The overall GC content is 37.1%, with the IR regions having a higher content (42.96%) and the LSC and SSC regions having lower content (34.93% and 32.02%, respectively). The genome encodes 129 genes, including 85 protein-coding genes, 36 tRNA genes, and 8 rRNA genes (Figure 2). Fourteen protein-coding genes are cis-splicing, including *rps*16, *atp*F, *rpo*C1, *ycf*3, *clp*P, *pet*B, *pet*D, *rpl*2 (×2), *ycf*15 (×2), *ndh*B (×2), and *ndh*A (Figure S2A). Two of these genes, *ycf*3 and *clp*P, encompass two introns. The *rps*12 gene is trans-splicing and includes two introns (Figure S2B). Five tRNA genes (*trn*K-UUU, *trn*G-UCC, *trn*L-UAA, *trn*I-GAU, and *trn*A-UGC) possess one intron.


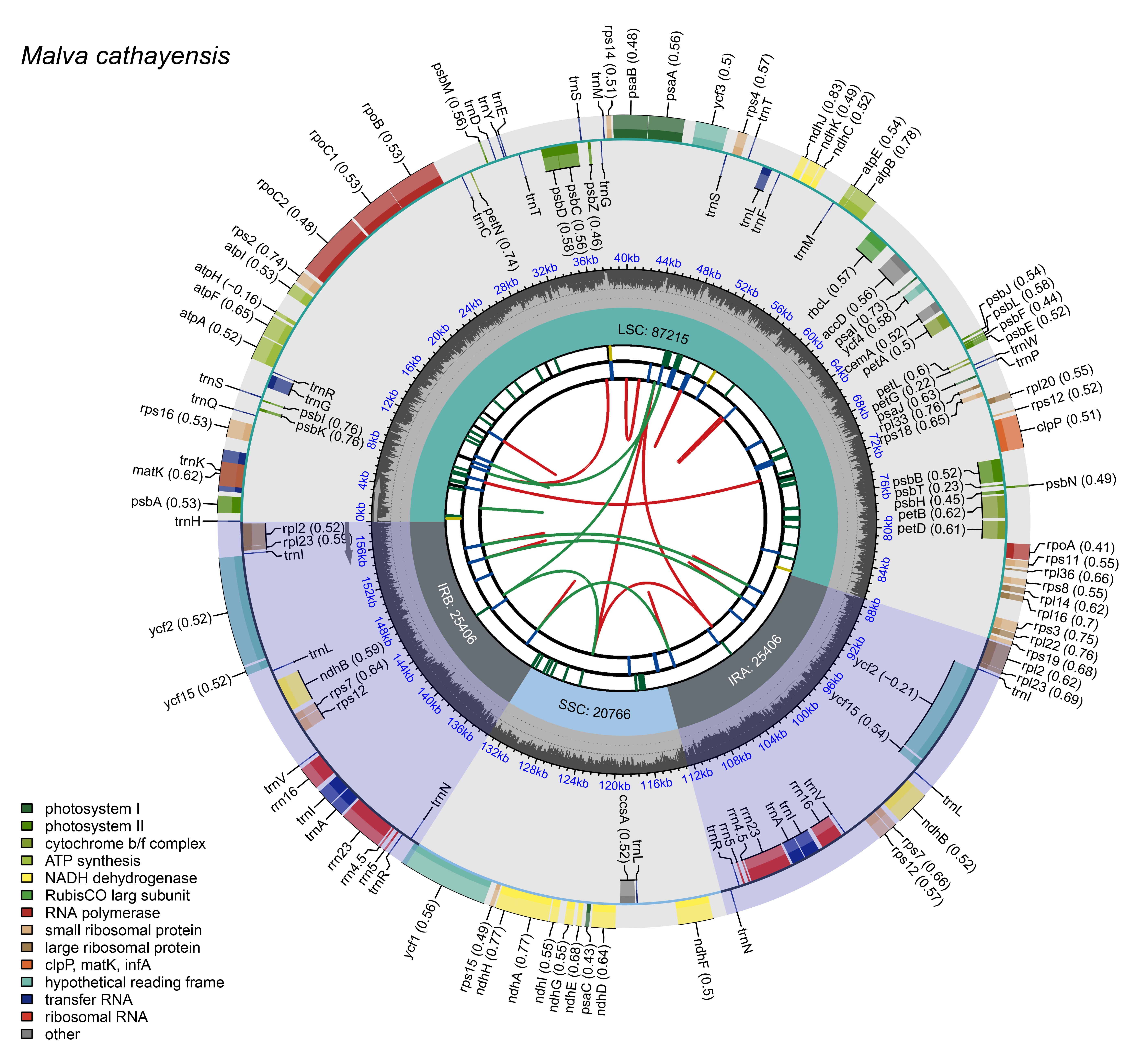


**Figure 2.** A schematic map depicting the overall features of the *Malva cathayensis* chloroplast genome.

From the center outward: the first track represents dispersed repeats, the second track displays long tandem repeats as short blue bars, and the third track shows short tandem repeats (microsatellites) as color-coded bars. The fourth track exhibits the small single-copy (SSC), inverted repeat (IRa and IRb), and large single-copy (LSC) regions. The fifth track plots the GC content of the genome, while the sixth track displays gene locations. The functional type of the genes is shown in the bottom left corner. For protein-coding genes, letters after gene names suggest functional subunits or family members, while numbers denote gene variants or functional differentiation. For ribosomal genes, numbers after gene names represent rRNA size in Svedberg units. Letters after gene names for tRNA genes denote the amino acids that the tRNA recognizes. For genes with unknown function, the numbers after the gene names correspond to distinct hypothetical coding genes.

Phylogenetic analysis using the ML method revealed that all *Malva* species clustered together two distinct clades (Figure 3). *Malva canariensis* and *M. wigandii* formed a monophyletic clade with 100% bootstrap support, while *M. cathayensis* grouped with *M. crispa*, *M. verticillata*, and *M. parviflora* in another monophyletic clade, with bootstrap values exceeding 90%.





**Figure 3.** The maximum likelihood phylogenetic tree featuring *Malva cathayensis*.

The tree was constructed using the complete chloroplast genome sequences of *M. cathayensis* (PP155498.2, this study) and five other *Malva* species, including *M. canariensis* (MT304828.1), *M. wigandii* (MT304827.1) (García-Mir *et al*., 2021), *M. crispa* (MZ327712.1), *M. verticillata* (MT083899.1, MT106775.1) (Li *et al*., 2020; Wang *et al*., 2020), and *M. parviflora* (MK860036.1) (Abdullah *et al*., 2020). *Abelmoschus esculentus* (OL348389.1) (Liu *et al*., 2023b) served as the outgroup. Bootstrap support values were calculated from 1000 replicates and are shown at each node. *Malva cathayensis* is highlighted in bold in the phylogenetic tree.

# Conclusion and Discussion

Our study presents the first complete chloroplast genome and the phylogenetic analysis of *M. cathayensis*, enhancing our understanding of its evolutionary relationships within the genus. It also provides a valuable genetic resource for future *Malva* genus research.

Comparative analyses showed that the chloroplast genomes of *Malva* species are highly conserved in structure, length, and gene content. The chloroplast genome of *M. cathayensis* has a quadripartite structure similar to that of other *Malva* species. The chloroplast genome of *M. cathayensis* is also comparable to other *Malva* species in terms of genome length and gene count, with a range of 158.1 kb to 158.5kb and 129 to 131 genes, respectively (Abdullah *et al*., 2020; García-Mir *et al*., 2021; Li *et al*., 2020; Wang *et al*., 2020).

The taxonomy and systematics of the genus *Malva* remain ambiguous and challenging due to the high level of homoplasy in morphological traits. A common approach divides *Malva* into two sections based on different criteria. Dalby and Malva (1968) proposed floral structure-based dividion. Ray (1995, 1998) described classification based on ITS molecular markers, fruit morphology, and seed structure. Jedrzejczyk and Rewers (2020) outlined the use of genome size estimation and ISSR molecular markers for categorization. *Malva parviflora* and *M. verticillata* are grouped in the same section of the phylogenetic tree created using chloroplast genome data, consistent with previous classifications based on different criteria (Ray and Malva, 1995, 1998; Jedrzejczyk and Rewers, 2020). The grouping of *M. cathayensis*, *M. crispa*, *M. parviflora* and *M. verticillata* in one section and *M. canariensis* and *M. wigandii* in another separate section offers valuable insights for resolving the taxonomy of *Malva*.

# Author contributions

The manuscript incorporates the contributions of all authors. Liqiang Wang conceptualized and designed this study. Liqiang Wang identified the species, and collected the sample. Junfei Liu and Weihan Yuan extracted the total DNA. Liqiang Wang assembled the chloroplast genome. Yuting Jiao annotated the chloroplast genome and Kaihua Zhang performed the phylogenetic analysis. Junfei Liu and Weihan Yuan analyzed the characteristics of the chloroplast genome. Shuming Zhang drafted the manuscript. All authors approved the publication of the version and agreed to be responsible for all aspects of the work.

# Funding

This work was supported by the Doctoral Fund Project of Heze University [XY20BS09] and Shandong Provincial Natural Science Foundation [ZR2021MC136].

# Disclosure statement

The authors declared no competing interests during the preparation and execution of this study. Dr. Liqiang Wang is the author of this manuscript and also serves as the Associate-Editor of the Mitochondrional DNA Part B journal. Dr. Liqiang Wang declared no conflicts of interest concerning the research findings in this study.

# Data availability statement

The chloroplast genome sequence has been deposited in GenBank (https://www.ncbi.nlm.nih.gov/genbank/) with the accession number of PP155498.2 (https://www.ncbi.nlm.nih.gov/nuccore/PP155498.2). The associated BioProject, Bio-Sample and SRA numbers are PRJNA928567, SAMN42702630 and SRR29906881.

# Ethics statement

The *Malva* *cathayensis* is not classified as an endangered species. It does not require specific permissions or licenses. In this study, the collection of *M. cathayensis* leaves was carried out in accordance with the guidelines provided by Heze University.

# References

Abdullah, Furrukh M, Iram S, *et al*. Correlations among oligonucleotide repeats, nucleotide substitutions, and insertion-deletion mutations in chloroplast genomes of plant family Malvaceae. Journal of Systematics and Evolution. 2020, 59(2): 388-402. doi: 10.1111/jse.12585.

Bolger AM, Marc L, Bjoern U. Trimmomatic: a flexible trimmer for Illumina sequence data. Bioinformatics. 2014, 30: 2114-2120. doi: 10.1093/bioinformatics/btu170.

Dalby DH, Malva L. Flora Europea. Rosaceae to Umbelliferae. Tutin TG, Heywood VH, Burges NA, Moore DM, Valentine DH, Walters SM, Weeb DA, Eds. Cambridge University Press: Cambridge, UK. 1968, 2: 249-251.

García-Mir L, Ojeda DI, Fuertes-Aguilar J. The complete chloroplast genome of *Malva wigandii* (Alef.) M.F. Ray (Malvaceae, Malvoideae). Mitochondrial DNA Part B Resources. 2021, 6(3): 1181-1182. doi: 10.1080/23802359.2021.1902409.

Gilbert MG, Tang Y, Dorr LJ. *Malva cathayensis* M.G. Gilbert, Y. Tang & Dorr. Folora of China. 2007, 12: 266.

Hafiza UR, Haq N, Rafia R, *et al*. Little mallow: A review of botany, composition, uses and biological potentials. International Journal of Chemical and Biochemical Sciences. 2017: 157-161.

Javad SR, Guiomar ML, Javier HÁA, *et al*. *Malva* species: Insights on its chemical composition towards pharmacological applications. Phytotherapy Research. 2020, 34(3): 546-567. doi: 10.1002/ptr.6550.

Jedrzejczyk I, Rewers M. Identification and genetic diversity analysis of edible and medicinal *Malva* species using flow cytometry and ISSR molecular markers. Agronomy. 2020, 10(5): 650. doi: 10.3390/agronomy10050650.

Jin J, Yu W, Yang J, *et al*. GetOrganelle: A fast and versatile toolkit for accurate *de novo* assembly of organelle genomes. Genome Biology. 2020, 21(1): 241. doi: 10.1186/s13059-020-02154-5.

Kalyaanamoorthy S, Minh BQ, Wong TKF, *et al*. ModelFinder: fast model selection for accurate phylogenetic estimates. Nature Methods. 2017, 14(6): 587-589. doi: 10.1038/nmeth.4285.

Katoh K, Standley DM. MAFFT multiple sequence alignment software version 7: improvements in performance and usability. Molecular Biology and Evolution. 2013, 30(4): 772-780. doi: 10.1093/molbev/mst010.

Li H. Minimap2: pairwise alignment for nucleotide sequences. Bioinformatics. 2018, 34(18): 3094-3100. doi: 10.1093/bioinformatics/bty191.

Li H, Handsaker B, Wysoker A, *et al*. The sequence alignment/map format and SAMtools. Bioinformatics. 2009, 25(16): 2078-2079. doi: 10.1093/bioinformatics/btp352.

Li RS, Liu J, Xu Li, *et al*. The complete chloroplast genome of *Malva verticillata* (Malvaceae). Mitochondrial DNA Part B Resources. 2020, 5(2): 1609-1610. doi: 10.1080/23802359.2020.1745106.

Liu S, Ni Y, Li J, *et al.* CPGView: A package for visualizing detailed chloroplast genome structures. Molecular Ecology Resources. 2023a, 23(3): 694-704. doi: 10.1111/1755-0998.13729.

Liu Y, Wang JY, Bai Y, *et al*. The whole chloroplast genome in *Abelmoschus esculentus* L. Moench. New Zealand Journal of Crop and Horticultural Science. 2023b, 51(1): 123-135. doi: 10.1080/01140671.2021.1960568.

Nguyen LT, Schmidt HA, von Haeseler A, *et al*. IQ-TREE: a fast and effective stochastic algorithm for estimating maximum-likelihood phylogenies. Molecular Biology and Evolution. 2015, 32(1): 268-274. doi: 10.1093/molbev/msu300.

Ray MF. New combinations in *Malva* (Malvaceae: Malveae). Novon. 1998, 3: 288-295. doi: 10.2307/3392022.

Ray MF. Systematics of *Lavatera* and *Malva* (Malvaceae, Malveae)—A new perspective. Plant Systematics and Evolution. 1995, 198: 29-53. doi: 10.1007/BF00985106.

Shi L, Chen H, Jiang M, *et al*. CPGAVAS2, an integrated plastome sequence annotator and analyzer. Nucleic Acids Research*.* 2019, 47(W1): W65-W73. doi: 10.1093/nar/gkz345.

Wang LR, Cai BL, Li JX, *et al*. The complete chloroplast genome sequence of *Malva verticillate*. Mitochondrial DNA Part B Resources. 2020, 5(2): 1669-1670. doi: 10.1080/23802359.2020.1742602.

Zhang S, Chen M, Li T, *et al*. A newly found cadmium accumulator--*Malva* *sinensis* Cavan. Journal of Hazardous Materials. 2010, 173(1-3): 705-709. doi: 10.1016/j.jhazmat.2009.08.142.


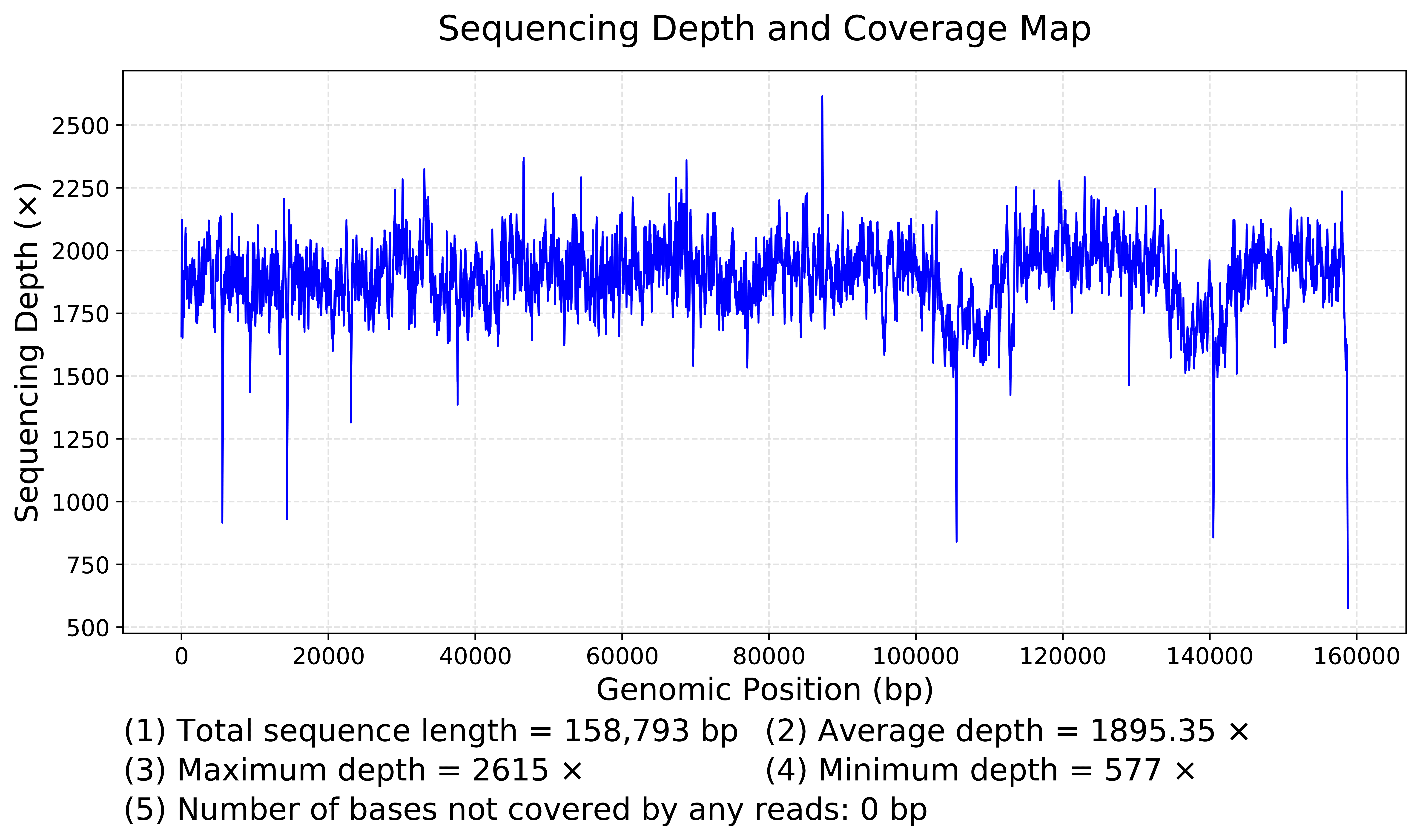


**Figure S1.** The sequencing depth of the complete *Malva cathayensis* chloroplast genome. The horizontal coordinate represents the nucleotide’s positional information. The vertical coordinate indicates the coverage depth at each position of the chloroplast genome, as determined using minimap2 (Li 2018) and samtools (Li *et al*. 2009).

A


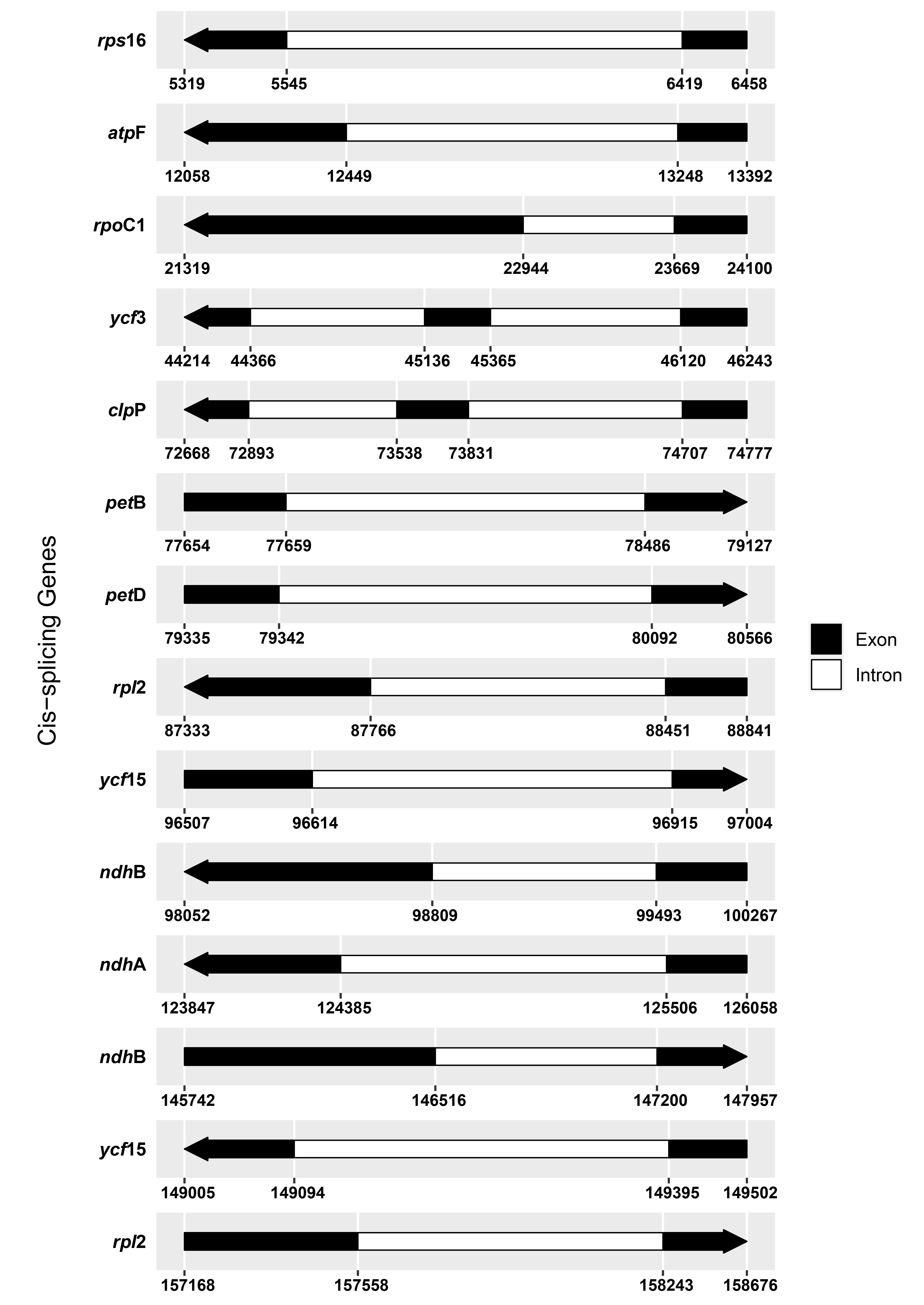

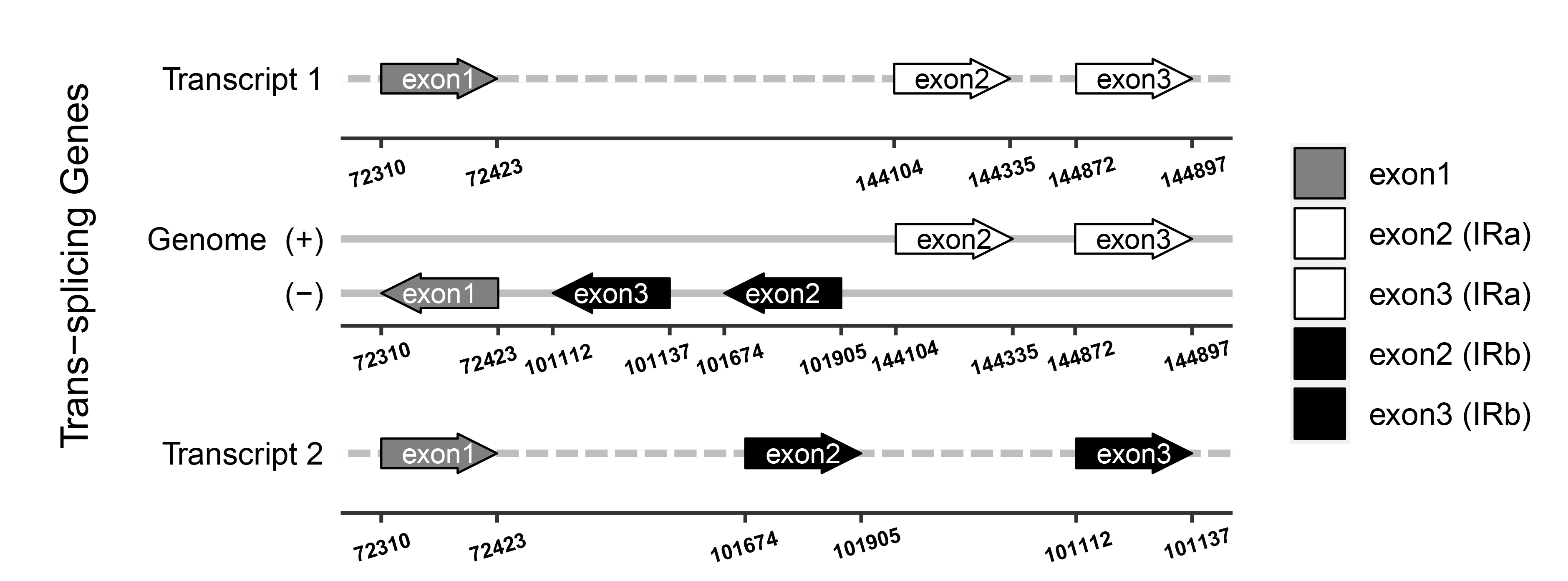


**A**

**B**

**Figure S2.** The *Malva cathayensis* chloroplast genome featuring 11 cis-splicing genes (A) and one trans-splicing gene, *rps*12 (B). The schematic diagrams were generated using CPGView software (Liu *et al*. 2023a).
